# Maternal high fat and/or salt consumption induces sex-specific inflammatory and nutrient transport in the rat placenta

**DOI:** 10.14814/phy2.12399

**Published:** 2015-05-19

**Authors:** Clare M Reynolds, Mark H Vickers, Claudia J Harrison, Stephanie A Segovia, Clint Gray

**Affiliations:** Liggins Institute and Gravida, National Centre for Growth and Development, University of AucklandAuckland, New Zealand

**Keywords:** Inflammation, maternal obesity, placenta

## Abstract

Maternal high fat and salt consumption are associated with developmental programming of disease in adult offspring. Inadequacies in placental nutrient transport may explain these ‘programmed effects’. Diet-induced inflammation may have detrimental effects on placental function leading to alteration of key nutrient transporters. We examined the effects of maternal high fat and/or salt diets on markers of placental nutrient transport and inflammation. Sprague–Dawley rats were assigned to (1) control (CD; 1% Salt 10% kcal from fat); (2) high salt (SD; 4% salt, 10% kcal from fat); (3) high fat (HF; 1% Salt 45% kcal from fat) or (4) high fat high salt (HFSD; 4% salt, 45% kcal from fat) 21 days prior to and throughout gestation. At embryonic day 18, dams were killed by isoflurane anesthesia followed by decapitation; placenta/fetuses were weighed, sexed, and collected for molecular analysis. Maternal SD, HF, and HFSD consumption decreased weight of placenta derived from male offspring; however, weight of placenta derived from female offspring was only reduced with maternal HF diet. This was associated with increased expression of LPL, SNAT2, GLUT1, and GLUT4 in placenta derived from male offspring suggesting increased fetal exposure to free fatty acids and glucose. Maternal SD, HF, and HFSD diet consumption increased expression of proinflammatory mediators IL-1*β*, TNF*α,* and CD68 in male placenta. Our results suggest that a proinflammatory placental profile results in detrimental alterations in nutrient transport which may contribute to the developmental origins of cardio-metabolic disturbances in offspring throughout life.

## Introduction

Over the past decade, global obesity rates have escalated to epidemic proportions, with alarming rates of childhood obesity observed in many Western countries (Berghofer et al. 2008; Ng et al. 2014). In modern Western societies the proportion of women of childbearing age presenting as overweight or obese is increasing (Chu et al. 2007) and represents about two-thirds of women in the US (Hillemeier et al. 2011). Evidence from both epidemiological and animal studies suggest that maternal obesity and unhealthy diet during pregnancy greatly increases the risk for complications such as gestational diabetes, preeclampsia, low and high birth weights and miscarriage (Denison et al. 2010; Alfaradhi and Ozanne 2011; Nodine and Hastings-Tolsma 2012). Furthermore, maternal obesity can enhance the risk for obesity and associated metabolic dysfunction in offspring, thereby perpetuating the cycle of obesity into the next generation (McMillen and Robinson 2005; Alfaradhi and Ozanne 2011; Li et al. 2013). In addition to increased consumption of saturated fats, “Western” diets are typically high in salt (Brown et al. 2009). Despite this, there are a paucity of data relating to the effects of increased salt on placental function and developmental programming of metabolic and cardiovascular disease, despite its relevance to contemporary Western diets.

The placenta ensures that the fetus, which represents a semiallogenic entity, is not subject to attack from the maternal immune system (Challis et al. 2009). Relatively minor abnormalities during placentation and its subsequent function are associated with pregnancy complications such as preterm birth and preeclampsia. Obesity is associated with a state of low-grade chronic inflammation. Increased nutritional supply promotes adipose tissue hypertrophy, which initiates a dysregulated immune response and stimulates migration of proinflammatory macrophages thereby initiating localized insulin resistance (Chawla et al. 2011; Lumeng and Saltiel 2011). This also promotes a systemic inflammatory profile with circulating proinflammatory cytokines, such as interleukin (IL)-1*β*, tumor necrosis factor (TNF)*α* and IL-6, free fatty acids (FFA) and triglycerides elevated in obese individuals (Stentz et al. 2004). There is recent evidence suggesting obesity-induced increases in circulating inflammatory factors promotes inflammation in the placenta and is associated with adverse pregnancy outcomes *via* dysregulation of nutrient transport to the fetus (Lager et al. 2014). Recent work by our group has shown that maternal obesity can lead to placental insufficiency and fetal and placental junctional zone growth restriction (Mark et al. 2011). A recent study from this group has shown that maternal high fat and/or salt intake during pregnancy alters maternal metabolic growth and food intake along with evidence of a detrimental effect on meta-inflammatory profiles and weanling offspring adiposity and insulin sensitivity (Reynolds et al. 2014).

In the present study, using an established model of maternal salt and/or moderate fat intake, we aimed to determine how maternal diets rich in saturated fatty acids and/or high in salt influence maternal inflammatory profiles, fetal growth, nutrient transfer and inflammation. Given that the placenta is derived from fetal cells we also aimed to determine whether or not placental gene expression was regulated in a sex-specific manner.

## Methods

### Animal experiments

All procedures described were approved by the Animal Ethics Committee at the University of Auckland (Approval R1069). Twenty-four female Sprague–Dawley rats were fed standard chow ad-libitum from weaning until day 90 and maintained at 25°C and a 12 h light: 12 h darkness cycle. Following the prepregnancy habituation period the experimental groups were fed either (1) Control (CD) purified standard diet (1% NaCl, 10% kcal from fat, *n* = 6); (2) 4% Salt diet (SD; 4% NaCl, 10% kcal from fat, *n* = 6); (3) High fat diet (HF; 1% NaCl, 45% kcal from fat, *n* = 6) or (4) High fat 4% Salt diet (HFSD; 4% NaCl, 45% kcal from fat, *n* = 6) for 21 days prior to mating (Table1). Female rats (110 days of age ± 5) were time-mated using an estrous cycle monitor (Fine Science Tools, Foster City, CA). Day 1 of pregnancy was determined by the presence of spermatozoa after a vaginal lavage and females individually housed thereafter. Pregnant animals were maintained on study diets throughout pregnancy. Food intake and body weight of dams were recorded every 2 days. Dams were culled at day 18 of gestation (E18; gestation in this strain is typically 22–23 days) (*n* = 6/group) by isoflurane anesthesia followed by decapitation. This time-point represents a period of rapid fetal growth, which may be relevant to the lower birthweights that are observed in HF-fed animals and thus relevant to the developmental programming of offspring health and disease paradigm. Trunk blood was collected in heparinized tubes and stored on ice until centrifugation and removal of plasma for analysis. All fetuses and placentas were collected, weighed, and sexed. One male and one female placenta from each litter were snap frozen for gene expression analysis. One male and one female placenta from each litter were fixed in 10% neutral-buffered formalin.

**Table 1:** Composition of experimental diets

	CD (D12450H)		SD (D13021101)		HF (D12451)		HFSD (D13021102)	
	gm	kcal	gm	kcal	gm	kcal	gm	kcal
Protein	19.2	20	18.5	20	23.7	20	22.8	20
Carbohydrate	67.3	70	64.8	70	41.4	35	39.9	35
Fat	4.3	10	4.1	10	23.6	45	22.7	45
Total		100		100		100		100
kcal/gm	3.85		3.70		4.73		4.55	
Ingredient								
Casein, 80 Mesh	200	800	200	800	200	800	200	800
L-Cystine	3	12	3	12	3	12	3	12
Corn Starch	452.2	1809	452.2	1809	72.8	291	72.8	291
Maltodextrin 10	75	300	75	300	100	400	100	400
Sucrose	172.8	691	172.8	691	172.8	691	172.8	691
Cellulose, BW200	50	0	50	0	50	0	50	0
Soybean Oil	25	225	25	225	25	225	25	225
Lard	20	180	20	180	177.5	1598	177.5	1598
Mineral Mix S10026	10	0	10	0	10	0	10	0
DiCalcium Phosphate	13	0	13	0	13	0	13	0
Calcium Carbonate	5.5	0	5.5	0	5.5	0	5.5	0
Potassium Citrate, 1 H_2_O	16.5	0	16.5	0	16.5	0	16.5	0
Sodium Chloride	10.1	0	41.3	0	9.9	0	33.1	0
Vitamin Mix V10001	10	40	10	40	10	40	10	40
Choline Bitartrate	2	0	2	0	2	0	2	0
Total	1055.05	4057	1096.35	4057	858.15	4057	891.25	4057

Diets were provided by Research Diets, New Jersey; USA.

### Materials

Primers and TaqMan Universal Mastermix were purchased from Applied Biosystems (ABI, Carlsbad, CA). All other reagents were purchased from Sigma Aldrich (Auckland, New Zealand) unless otherwise stated.

### Plasma analysis

Plasma insulin (Crystal Chem Inc, Downers Grove, IL), IL-1*β*, and TNF*α* concentrations (Quantikine kits; R&D Systems Europe, Abingdon, UK) were measured enzymatically. Triacylglyceride (TAG), analysis was performed using an enzymatic colorimetric assay on a Hitachi 902 autoanalyzer (Hitachi High Technologies Corporation, Tokyo, Japan).

### Gene expression analysis

RNA was extracted from whole placentas using TRI-Reagent (50 mg tissue/mL) and stored at −80°C. Single-stranded cDNA was prepared using High-Capacity cDNA Archive Kit (Applied Biosystems, Warrington, UK). mRNA expression was quantified by real-time PCR (RT-PCR) on an ABI 7700 Sequence Detection System (Perkin-Elmer Applied Biosystems). TaqMan real-time PCR was performed for IL-1*β*, TNF*α*, CD68, lipoprotein lipase (LPL), CD36, glucose transporter (GLUT)1, GLUT4, system N/A amino acid transporter (SNAT)2, SNAT4 and delta homolog (DLK)1 using Pre-Developed Assay Reagent Kits. To control for between-sample variability, mRNA levels were normalized to the geometric mean of cyclophilin A and Hypoxanthine Phosphoribosyltransferase (HPRT) for each sample by subtracting the *C*_t_ of controls from the *C*_t_ for the gene of interest producing a Δ*C*_t_ value. The Δ*C*_t_ for each treatment sample was compared to the mean Δ*C*_t_ for control samples using the relative quantification 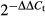 method to determine fold-change (Livak and Schmittgen 2001).

### Hematoxylin and Eosin staining

Placental samples were immediately fixed in 10% neutral-buffered formalin and paraffin embedded. Sections were prepared (5 *μ*m) using Leica EG1150H Machine. Sections were stained for Hematoxylin and Eosin (H&E) using standard procedures. Sections were analyzed under light microscope (Nikon 800, Tokyo, Japan) and images taken (Nikon FDX-35, Tokyo, Japan) and processed with NIS Elements-D software (Nikon). Placental zone analysis was performed using ImageJ software analysis at magnifications (×1.25) with four sections per placenta).

### Statistics

Statistical analysis was performed using SigmaPlot for Windows version 12.0 (Systat Software Inc., San Jose, CA). All data were analyzed by three-way factorial ANOVA, with maternal high fat, maternal high salt intake and sex as factors, results are displayed in boxes above graphs and interactions are presented in the text. Holm-Sidak post hoc tests were performed to detect any differences between groups, these are depicted as *P < 0.05. Differences were considered significant at *P* < 0.05. All data are presented as means ± SEM.

## Results

### Effects of maternal high fat and/or high salt diet on weight and systemic inflammation

HF and HFSD groups had increased body weight compared to CD and SD at embryonic day 18 (Table2). Furthermore, HF dams had increased fasting plasma insulin, IL-1*β* and TNF*α* concentrations compared to CD and SD indicating that these treatments induced a state of low-grade inflammation in line with the initiation of an insulin-resistant phenotype, as outlined in a previous manuscript (Reynolds et al. 2014). While a trend toward increased concentrations was observed with HFSD dams, only TNF*α* reached significance (Table2). TAG was increased in response to SD and HFSD compared to CD and HF diets (Table2) demonstrating potential dysregulation of lipid homeostasis in these dams.

**Table 2:** Effect of maternal diet on maternal weight and plasma profiles

	CD	SD	HF	HFSD
Weight (g)†	418 ± 16.9	449.7 ± 8.8	468.5 ± 9.7*	461.9 ± 19*
IL-1 *β* (pg/mL)†	8.9 ± 0.3	8.1 ± 1.6	16.5 ± 2.7*	12.4 ± 3.5
TNF*α* (pg/mL)†	3.7 ± 2.6	2.7 ± 1.7	10.5 ± 3.1*	11.3 ± 3.1*
Insulin (ng/mL)	1.0 ± 0.2	0.85 ± 0.2	1.7 ± 0.5*	1.3 ± 0.2
TAG (mmol/L)#	3.15 ± 0.35	4.56 ± 0.32*	3.04 ± 0.37	4.23 ± 0.51*

† *P* < 0.05 represents high fat diet-induced effects

# *P* < 0.05 represents salt effects

* *P* < 0.05 w.r.t. CD, *n* = 6 per group.

Data are presented as mean ± SEM. All intra-assay variations had a CV of <10%.

### Effects of maternal high fat and/or high salt diet on placental morphology

**Figure 1:**
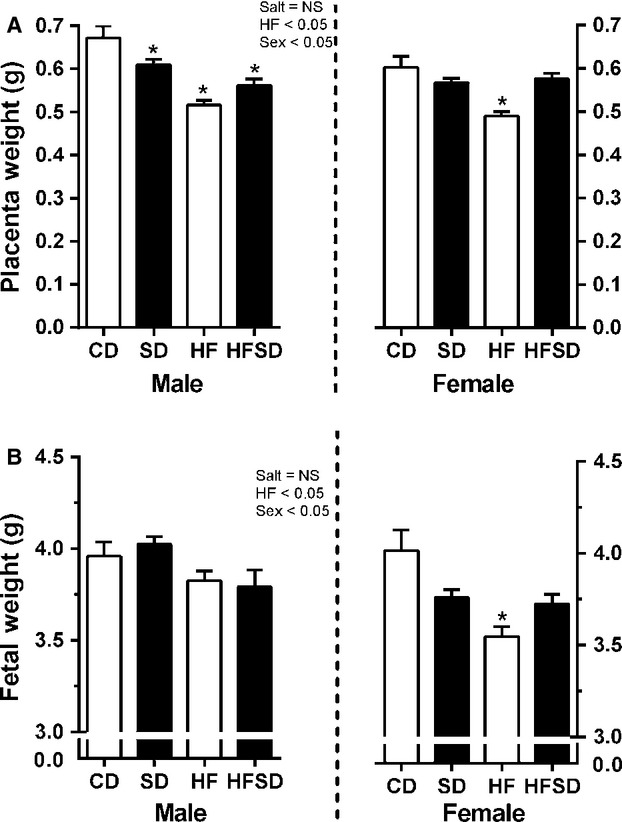
Placental and fetal weights: Placentas and fetuses were collected, sexed, and weighed. Data are expressed as means ± SEM. (*P < 0.05 w.r.t CD, *n* = 6 L).

As maternal metabolic and inflammatory profiles can be associated with placental dysfunction, placental weights and morphology were assessed. There was no difference in litter size between groups. In male placentas there was a significant HF effect with decreased placental weight. This was accompanied by an effect for sex (Salt × Fat × Sex interaction). Post hoc analysis determined decreases in SD, HF, and HFSD compared to CD groups (Fig.1A). This was associated with a sex-specific effect of HF effect on male fetal weight (Fig.1B; Salt × Fat × Sex interaction). In female fetuses and placentas, an HF effect was accompanied by an interaction between maternal salt and fat, however, post hoc analysis determined only HF significantly decreased fetal and placental weight (Fig.1A and B).

**Figure 2:**
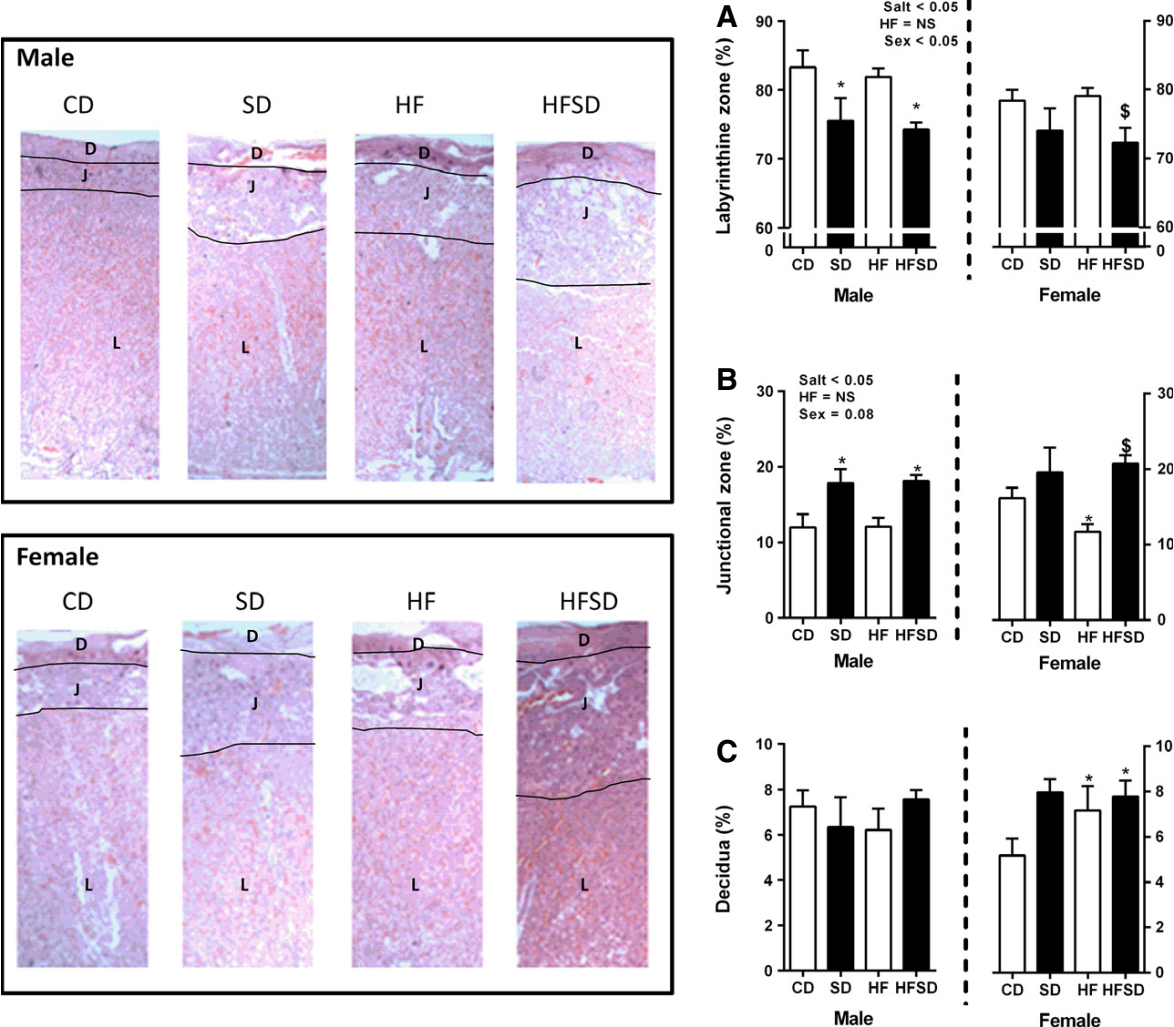
Distribution of placental layers: Data are expressed as mean percentage of total thickness for (A) labyrinthine zone, (B) junctional zone, and (C) decidual zone. Representative images are displayed. Data are expressed as means ± SEM. (*P < 0.05 w.r.t CD; ^$^*P* < 0.05 w.r.t HF, *n* = 6 males; *n* = 6 females from independent litters).

Abnormal distribution of placental layers can be indicative of pathology, we therefore histologically examined junction, labyrinthine and decidual zone areas. There was a reduction in labyrinthine zone size in both male and female salt groups, post hoc significance was attained in male SD and HFSD and female HFSD groups. There was no difference between CD and HF groups (Fig.2A). There was an effect of salt on junctional zone size accompanied by a significant increase in SD and HFSD male placentas and HFSD female placentas. There was a decrease in HF compared to CD in female placentas (Fig.2B). While there was no difference in decidua area in male placentas there were significant increases in female SD, HF, and HFSD groups compared to CD (Fig.2C).

### Sex-specific effects of maternal diet on placental inflammation

**Figure 3:**
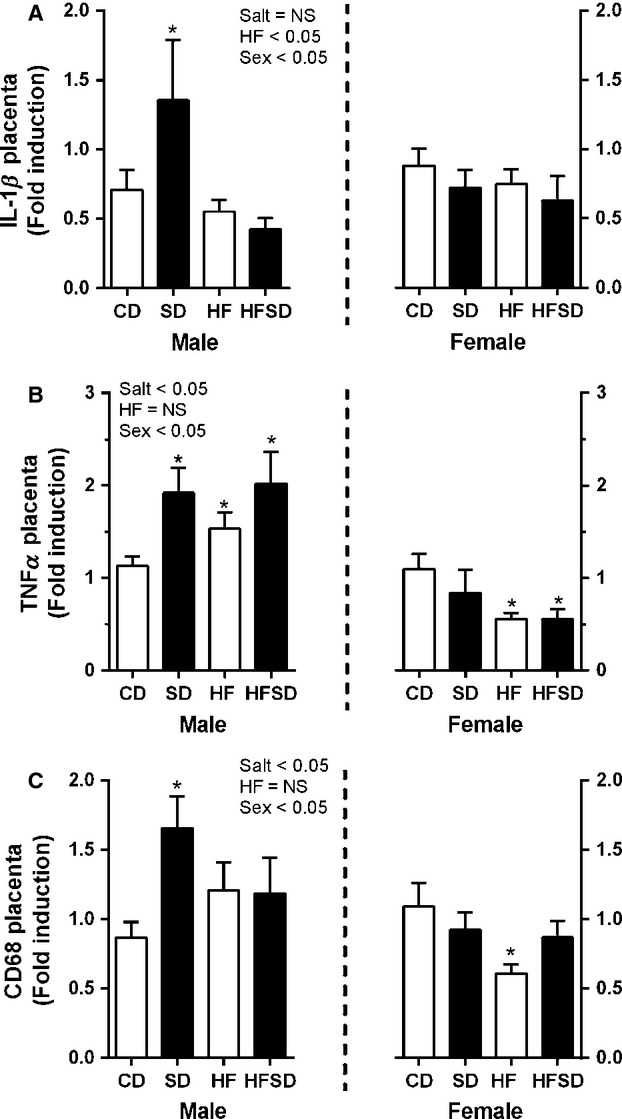
Placental inflammatory gene expression: Placental mRNA expression of (A) IL-1*β*, (B) TNF*α*, and (C) CD68 was analyzed by RT-PCR *P < 0.05 w.r.t CD; *n* = 6 males; *n* = 6 females from independent litters). Values are expressed as means ± SEM.

Given the increase in maternal systemic inflammation, we opted to examine the expression of inflammatory markers in the placenta. Maternal high salt diet-induced upregulation of inflammatory cytokines IL-1*β* and TNF*α* along with the macrophage marker CD68 in male placentas (HF diet × salt × sex interaction *P* < 0.05) with post hoc tests determining significance in SD groups, however, only TNF*α* was significant with HF and HFSD placentas (Fig.3). Conversely, high fat diet-induced downregulation of TNF*α* and CD68 was observed in female placentas (HF diet × sex interaction *P* < 0.05). There was no difference in IL-1*β* gene expression in female placentas (Fig.3).

### Sex-specific effects of maternal diet on placental nutrient transporters

**Figure 4:**
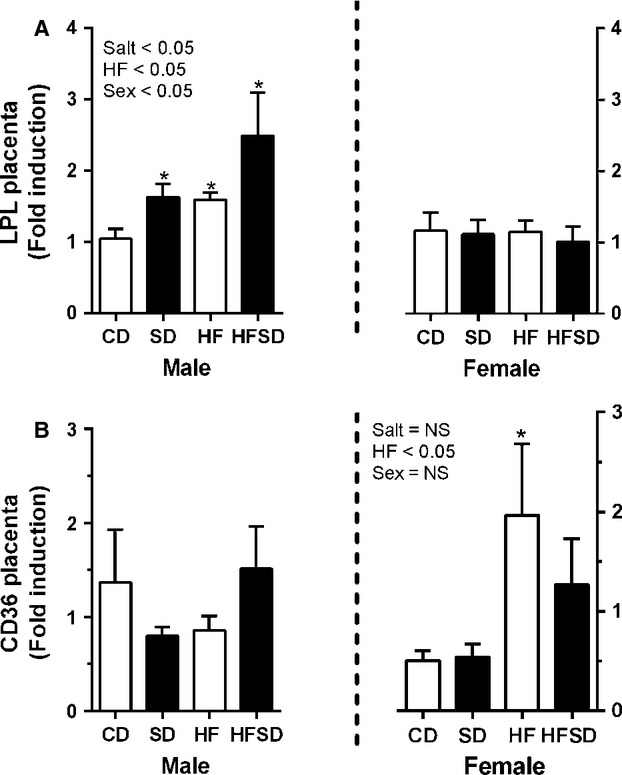
Placental lipid transport gene expression: Placental mRNA expression of (A) LPL, (B) CD36 was analyzed by RT-PCR (*P < 0.05 w.r.t CD; *n* = 6 males; *n* = 6 females from independent litters). Data are expressed as means ± SEM.

**Figure 5:**
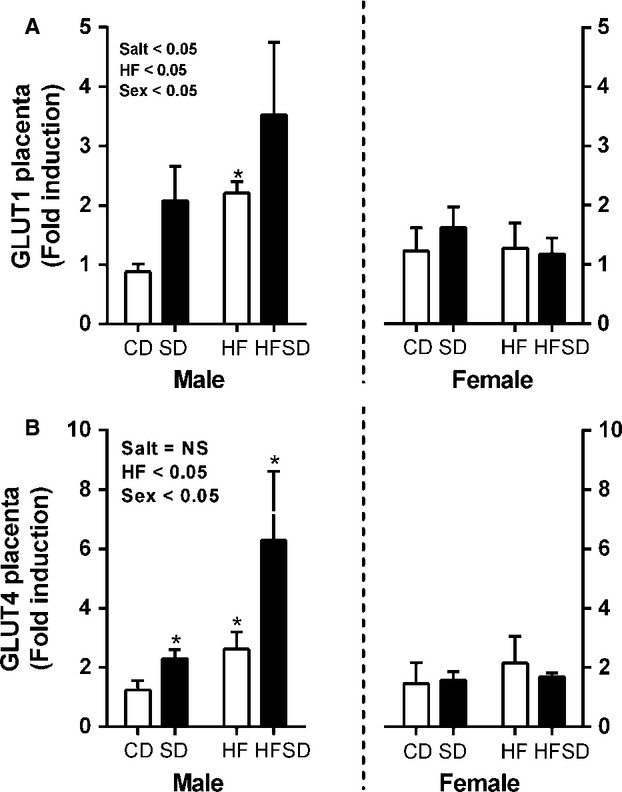
Placental glucose transporter gene expression: Placental mRNA expression of (A) GLUT1, (B) GLUT4 was analyzed by RT-PCR (*P < 0.05 w.r.t CD; *n* = 6 males; *n* = 6 females from independent litters). Data are expressed as means ± SEM.

Disruption of placental nutrient transport can have serious effects on fetal development and growth, we therefore examined RNA expression of lipid, glucose, and amino acid transport. There was a significant effect of maternal salt, maternal high fat diet, and offspring sex on LPL expression with increased expression in male placenta from SD, HF, and HFSD groups, female placenta displayed no difference between groups (Fig.4A). CD36, a fatty acid transporter, was also examined while there was no difference in male placentas, there was increased expression in HF female placentas, however, no overall effect of sex was observed (Fig.4B). GLUT1 and GLUT4 were also examined. There was no difference between groups with female placentas. However, there was a significant effect of maternal salt, maternal fat and offspring sex with increased expression in HF and HFSD male placentas (Fig.5A and B).

**Figure 6:**
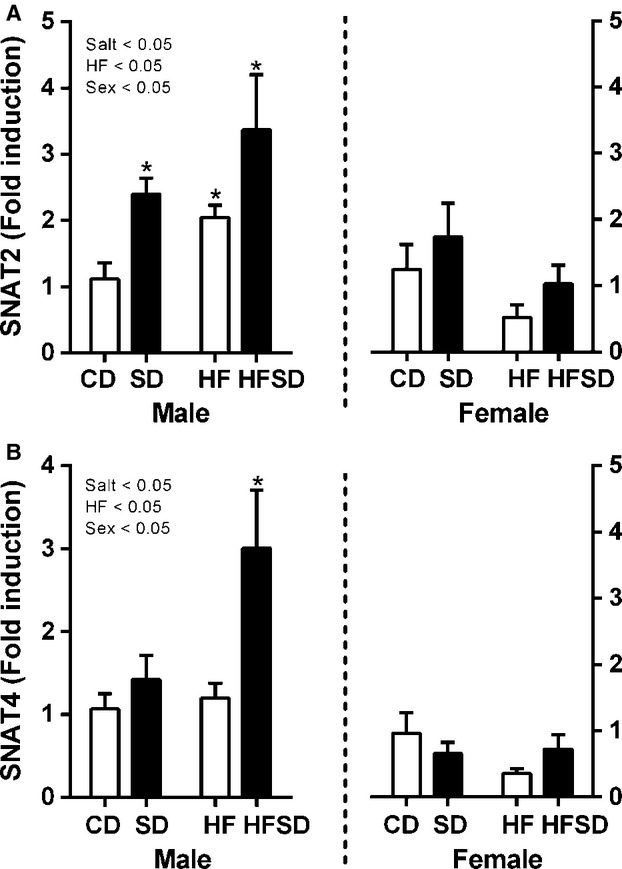
Placental amino acid transporter gene expression: Placental mRNA expression of (A) SNAT2, (B) SNAT4 was analyzed by RT-PCR (*P < 0.05 w.r.t CD; *n* = 6 males; *n* = 6 females from independent litters). Data are expressed as means ± SEM.

Sex-specific effects were observed with no difference in amino acid transporters SNAT2 and SNAT4 in placentas from female offspring. There was an increase in SD, HF, and HFSD expression of SNAT2 (Salt × HF × Sex interaction; *P* < 0.05), however, increased expression of SNAT4 was only observed in HFSD male placenta compared to CD (Salt × HF × Sex Interaction; Fig.6).

**Figure 7:**
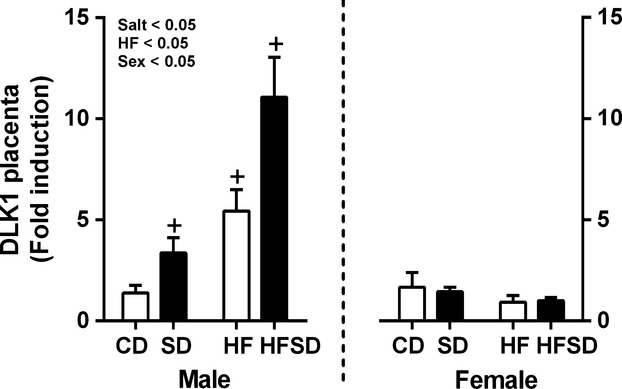
Placental DLK1 gene expression: Placental mRNA expression of DLK1 was analyzed by RT-PCR (*P < 0.05 w.r.t CD; *n* = 6 males; *n* = 6 females from independent litters). Data are expressed as means ± SEM.

DLK1 has been implicated in the regulation of nutrient transport between mother and fetus, overexpression is also thought to interfere with placental zone morphology. Given alterations in placental nutrient transporter expression and placental zone morphology in treatment groups we examined its expression. There was a maternal salt, maternal HF, and offspring sex effect, with increased expression in male SD, HF, and HFSD placentas compared to CD. There was no difference between groups with female placentas (Fig.7).

## Discussion

There is evidence that low- and high-birth weights can predict a wide array of adult onset conditions including obesity, metabolic, and cardiovascular disease (Barker 2007; Gluckman et al. 2008). Maternal insults such as over- or undernutrition and stress during critical points of development can have profound effects on the development of important biological systems such as the immune system (Reynolds et al. 2013), the vasculature (Hanson and Gluckman 2011) and hormonal regulation (Vickers 2007; Vieau et al. 2007) resulting in detrimental long-term effects on the offspring. Interestingly, these effects often demonstrate marked variation between the sexes (Gabory et al. 2013). As the main barrier between mother and fetus, placental adaptability is thought to play a major role in developmental programming and potential vertical transmission of metabolic and cardiovascular disease risk. Given that the placenta is traditionally viewed as an asexual organ, the sex of the placenta is overlooked in many studies. However, considering the abundance of sexually dimorphic responses to maternal nutrition within the developmental programming paradigm, it is reasonable to assume that the placenta also plays a major role in physiologic responses to maternal nutritional status and these responses are also largely sex specific. As described previously, and in the current study, we observe that consumption of a high fat diet is associated with decreased placental weight in both males and females (Thornburg et al. 2010). However, decreased placental weight in response to maternal salt intake was only observed with male offspring. Whilst a reduction in fetal weight is only observed in response to a maternal high fat diet, several human studies have demonstrated an association between placental weight and later life disease with individuals on the lower and higher end of the weight spectrum (Barker et al. 1990, 2010; Thornburg et al. 2010).

Disruption of placental organization during pregnancy is a causative factor in fetal growth restriction and is also a feature of pregnancy disorders such as preeclampsia (Kim et al. 2014). The placental labyrinth zone is the largest proportion of the rodent placenta and is primarily responsible for the transfer of nutrients and waste products between mother and fetus. In the current study there was no effect of maternal HF on labyrinth zone area; whilst maternal salt resulted in reduced labyrinth zone area in males only. The junctional zone is predominantly populated with trophoblast giant cells and spongiotrophoblasts; these cells represent a major source of endocrine activity within the placenta and exhibit the capability to directly influence fetal growth. Previous work by our group has demonstrated that maternal HF diet results in reduced junctional zone weight (Mark et al. 2011). This was replicated in the current study, where female fetuses had reduced junctional zone area correlating with reduced fetal size. Interestingly, there is a significant increase in junctional zone area in response to maternal salt intake in both male and female placenta. Given the role of this zone in hormonal regulation of fetal growth, increased junctional zone area may explain the differences in relative size of salt compared to HF exposed placenta and fetuses.

Obesity is associated with a state of chronic low-grade inflammation (Hotamisligil 2006). As such we demonstrate increased systemic cytokine concentrations in dams exposed to high fat diets (Reynolds et al. 2014). While inflammatory processes are essential for normal pregnancy progression and maintenance, dysregulation of immune function is a major contributor to pregnancy-related disorders (Challis et al. 2009; Denison et al. 2010). Despite no evidence of increased maternal circulating cytokines, placentas associated with male SD offspring display a pronounced inflammatory gene expression profile with enhanced IL-1*β* and TNF*α* expression. This is accompanied by an increase in the expression of macrophage marker CD68, indicating progressive macrophage infiltration into the placenta. Notably, there is increased TNF*α* expression in HF and HFSD male placentas. Contrary to our findings in placenta from male fetuses, there are no salt-induced increases in inflammatory gene expression in female placenta and there is a maternal HF-induced dampening of both TNF*α* and CD68 expression. The presence of enhanced inflammatory gene expression and evidence of macrophage infiltration in SD groups in the absence of maternal obesity or systemic inflammation may be indicative of an underlying pathology, which, despite going unnoticed in the maternal circulation, contributes to fetal programming. It is possible that alteration of placental inflammatory factors may influence programming of the immune system of male offspring thereby contributing to low-grade inflammation associated with the risk of obesity and metabolic dysfunction in later life, by which male offspring are more at risk than their female siblings.

Given the shifts in placental zone area between maternal diets, we examined the expression of several key genes related to placental growth, fatty acid and glucose uptake. Dlk1 belongs to the Delta-Notch family of signaling molecules and is highly expressed in both the placenta and developing fetus (Yevtodiyenko and Schmidt 2006). It is thought to play a role in the regulation of growth factors and maternal fetal nutrient transfer. Furthermore, there is evidence that increased Dlk1 interferes with morphogenesis of the placental labyrinth zone. Therefore, increased expression of Dlk1 in response to maternal salt and HF diets may contribute to aberrant placental structure and placental insufficiency. In addition, placenta from maternal salt and fat exposed male fetuses display increased LPL, a lipase enzyme which hydrolyzes triglycerides catalyzing one of the initial steps in placental fatty acid transport (Goldberg and Merkel 2001). Coupled with evidence of increased gestational hyperlipidemia in salt-fed dams this suggests enhancement of placental lipid accumulation in these animals. This may, in part, contribute to the enhanced inflammatory profile in SD animals thus exacerbating placental dysfunction. However, there is conflicting evidence regarding the role of increased LPL expression in terms of fetal growth. In pregnancies complicated by extreme growth restriction there is evidence of increased LPL expression, however, it is noteworthy that this does not relate to LPL activity (Tabano et al. 2006). There is also evidence demonstrating that increased lipid availability results in fetal overgrowth (Magnusson et al. 2004; Magnusson-Olsson et al. 2006). While there is no evidence of altered expression of the fatty acid transporter CD36 in male placentas there are increases in HF exposed female fetuses indicating increased uptake of fatty acids in the placenta and increased supply to the fetus. These sex-specific effects may, at least in part, allude to differential mechanisms of fetal growth restriction in male and female offspring. Interestingly, increased LPL expression is accompanied by increased expression of glucose transporters, GLUT4 and GLUT1. As glucose is the main fuel for both placenta and fetus, growth and function are heavily reliant on GLUT function. Expression of System A sodium dependant glucose transporters, SNAT2 and SNAT4 was also measured. These transporters are critical for uptake of both nonessential and essential amino acids and are well characterized in relation to fetal growth (Jansson et al. 2002; Roos et al. 2009). While recent studies have demonstrated increased amino acid transporter expression in placenta of dams exposed to HFD, these pregnancies resulted in fetal overgrowth rather than fetal growth restriction (Jones et al. 2009). It is thought that in cases of placental insufficiency the placenta overcompensates and may increase expression of genes relating to nutrient transport. However, as pregnancy advances these compensatory mechanisms are insufficient to support the growing fetus and growth restriction occurs (Lager and Powell 2012). Given previous evidence from this group (Mark et al. 2011) we therefore speculate that aberrant placental morphology may represent a potential compensatory mechanism to prevent fetal overgrowth induced by the seemingly increased nutrient availability in salt-exposed male placentas.

While sex-specific differences in adult offspring can be explained by hormonal differences, these effects in embryos and neonates are not as clear cut. There is evidence that sex differences are evident prior to gonadal development. It is thought that these differences may stem from alterations in sex chromosome number as male and female embryos only differ in sex chromosome content. While X-chromosome inactivation equalizes X-linked expression in adults this process occurs later in development resulting in higher X-linked gene expression in female embryos. This is also known to affect autosomal gene expression. In addition imprinting with preferential expression of paternal alleles may also induce differential sex-specific gene expression. Several metabolic genes are known to be influenced by X-dosage and imprinting, and influence the ability of the embryo to respond to maternal metabolic or environmental stressors. While not examined in this current study, it is likely that the sex-specific effects observed stem from a combination of X-dosage and imprinting effects.

In conclusion, the current study highlights the impact of moderate maternal salt and fat intakes on placental structure and expression of key determinants of fetal growth. Alterations in placental structure indicate that even moderate consumption of typically “Western” diet components may alter placental architecture in a manner indicative of placental insufficiency. Increased expression of glucose and fatty acid transporters suggests an attempt to compensate for this diet-induced placental insufficiency. While maternal obesity and increased saturated fatty acid intakes are typically associated with placental dysfunction and fetal growth restriction, this study demonstrates substantial inflammatory gene expression in conjunction with an altered placental morphology in the absence of overt maternal meta-inflammatory dysfunction. The present study has highlighted some of the possible pathways that underpin developmental programming and the sex-specific phenotypic traits that are consistently observed across numerous studies. Previously published work from our group and work from others add to the ongoing body of evidence that may, in part, explain the developmental origins of later life disease. Specifically, the sex differences frequently observed within these studies may be driven by sexually dimorphic placental alterations which occur as a result of maternal gestational diets.

## References

[b1] Alfaradhi MZ, Ozanne SE (2011). Developmental programming in response to maternal overnutrition. Front. Genet.

[b2] Barker DJ (2007). The origins of the developmental origins theory. J. Intern. Med.

[b3] Barker DJ, Bull AR, Osmond C, Simmonds SJ (1990). Fetal and placental size and risk of hypertension in adult life. BMJ.

[b4] Barker DJ, Thornburg KL, Osmond C, Kajantie E, Eriksson JG (2010). The surface area of the placenta and hypertension in the offspring in later life. Int. J. Dev. Biol.

[b5] Berghofer A, Pischon T, Reinhold T, Apovian CM, Sharma AM, Willich SN (2008). Obesity prevalence from a European perspective: a systematic review. BMC Public Health.

[b6] Brown IJ, Tzoulaki I, Candeias V, Elliott P (2009). Salt intakes around the world: implications for public health. Int. J. Epidemiol.

[b7] Challis JR, Lockwood CJ, Myatt L, Norman JE, Petraglia JF, Strauss F (2009). Inflammation and pregnancy. Reprod. Sci.

[b8] Chawla A, Nguyen KD, Goh YP (2011). Macrophage-mediated inflammation in metabolic disease. Nat. Rev. Immunol.

[b9] Chu SY, Callaghan WM, Kim SY, Schmid CH, Lau J, England LJ (2007). Maternal obesity and risk of gestational diabetes mellitus. Diabetes Care.

[b10] Denison FC, Roberts KA, Barr SM, Norman JE (2010). Obesity, pregnancy, inflammation, and vascular function. Reproduction.

[b11] Gabory A, Roseboom TJ, Moore T, Moore LG, Junien C (2013). Placental contribution to the origins of sexual dimorphism in health and diseases: sex chromosomes and epigenetics. Biol. Sex Differ.

[b12] Gluckman PD, Hanson MA, Cooper C, Thornburg KL (2008). Effect of in utero and early-life conditions on adult health and disease. N. Engl. J. Med.

[b13] Goldberg IJ, Merkel M (2001). Lipoprotein lipase: physiology, biochemistry, and molecular biology. Front Biosci.

[b14] Hanson M, Gluckman P (2011). Developmental origins of noncommunicable disease: population and public health implications. Am. J. Clin. Nutr.

[b15] Hillemeier MM, Weisman CS, Chuang C, Downs DS, McCall-Hosenfeld J, Camacho F (2011). Transition to overweight or obesity among women of reproductive age. J. Womens Health (Larchmt).

[b16] Hotamisligil GS (2006). Inflammation and metabolic disorders. Nature.

[b17] Jansson T, Ekstrand Y, Bjorn C, Wennergren M, Powell TL (2002). Alterations in the activity of placental amino acid transporters in pregnancies complicated by diabetes. Diabetes.

[b18] Jones HN, Woollett LA, Barbour N, Prasad PD, Powell TL, Jansson T (2009). High-fat diet before and during pregnancy causes marked up-regulation of placental nutrient transport and fetal overgrowth in C57/BL6 mice. FASEB J.

[b19] Kim DW, Young SL, Grattan DR, Jasoni CL (2014). Obesity during pregnancy disrupts placental morphology, cell proliferation, and inflammation in a sex-specific manner across gestation in the mouse. Biol. Reprod.

[b20] Lager S, Powell TL (2012). Regulation of nutrient transport across the placenta. J. Pregnancy.

[b21] Lager S, Samulesson AM, Taylor PD, Poston L, Powell TL, Jansson T (2014). Diet-induced obesity in mice reduces placental efficiency and inhibits placental mTOR signaling. Physiol. Rep.

[b22] Li M, Reynolds CM, Sloboda DM, Gray C, Vickers MH (2013). Effects of taurine supplementation on hepatic markers of inflammation and lipid metabolism in mothers and offspring in the setting of maternal obesity. PLoS ONE.

[b23] Livak KJ, Schmittgen TD (2001). Analysis of relative gene expression data using real-time quantitative PCR and the 2(-Delta Delta C(T)) Method. Methods.

[b24] Lumeng CN, Saltiel AR (2011). Inflammatory links between obesity and metabolic disease. J. Clin. Invest.

[b25] Magnusson-Olsson AL, Hamark B, Ericsson A, Wennergren M, Jansson T, Powell TL (2006). Gestational and hormonal regulation of human placental lipoprotein lipase. J. Lipid Res.

[b26] Magnusson AL, Waterman IJ, Wennergren M, Jansson T, Powell TL (2004). Triglyceride hydrolase activities and expression of fatty acid binding proteins in the human placenta in pregnancies complicated by intrauterine growth restriction and diabetes. J. Clin. Endocrinol. Metab.

[b27] Mark PSC, Connor K, Patel R, Vickers M, Waddell B, Sloboda D (2011). A maternal high fat diet in the rat reduces fetal and placental junctional zone weights and increases placental expression of PPAR*γ* and total VEGFa. J. Dev. Orig. Health. Dis.

[b28] McMillen IC, Robinson JS (2005). Developmental origins of the metabolic syndrome: prediction, plasticity, and programming. Physiol. Rev.

[b29] Ng M, Fleming T, Robinson M, Thomson B, Graetz N, Margono C (2014). Global, regional, and national prevalence of overweight and obesity in children and adults during 1980–2013: a systematic analysis for the Global Burden of Disease Study 2013. Lancet.

[b30] Nodine PM, Hastings-Tolsma M (2012). Maternal obesity: improving pregnancy outcomes. MCN Am. J. Matern. Child Nurs.

[b31] Mark PJ, Sisala CS, Connor K, Patel R, Lewis JL, Vickers MH (2011). A maternal high-fat diet in rat pregnancy reduces growth of the fetus and the placental junctional zone, but not placental labyrinth zone growth. J. Dev. Orig. Health Dis.

[b32] Reynolds CM, Li M, Gray C, Vickers MH (2013). Pre-weaning growth hormone treatment ameliorates bone marrow macrophage inflammation in adult male rat offspring following maternal undernutrition. PLoS ONE.

[b33] Reynolds CM, Vickers MH, Harrison CJ, Segovia SA, Gray C (2014). High fat and/or high salt intake during pregnancy alters maternal meta-inflammation and offspring growth and metabolic profiles. Physiol. Rep.

[b34] Reynolds CM, Vickers MH, Harrison CJ, Segovia SA, Gray C (2014). High fat and/or high salt intake during pregnancy alters maternal meta-inflammation and offspring growth and metabolic profiles. Physiol. Rep.

[b35] Roos S, Lagerlof O, Wennergren M, Powell TL, Jansson T (2009). Regulation of amino acid transporters by glucose and growth factors in cultured primary human trophoblast cells is mediated by mTOR signaling. Am. J. Physiol. Cell Physiol.

[b36] Stentz FB, Umpierrez GE, Cuervo R, Kitabchi AE (2004). Proinflammatory cytokines, markers of cardiovascular risks, oxidative stress, and lipid peroxidation in patients with hyperglycemic crises. Diabetes.

[b37] Tabano S, Alvino G, Antonazzo P, Grati FR, Miozzo M, Cetin I (2006). Placental LPL gene expression is increased in severe intrauterine growth-restricted pregnancies. Pediatr. Res.

[b38] Thornburg KL, O'Tierney PF, Louey S (2010). Review: The placenta is a programming agent for cardiovascular disease. Placenta.

[b39] Vickers MH (2007). Developmental programming and adult obesity: the role of leptin. Curr. Opin. Endocrinol. Diabetes Obes.

[b40] Vieau D, Sebaai N, Leonhardt M, Dutriez-Casteloot I, Molendi-Coste O, Laborie C (2007). HPA axis programming by maternal undernutrition in the male rat offspring. Psychoneuroendocrinology.

[b41] Yevtodiyenko A, Schmidt JV (2006). Dlk1 expression marks developing endothelium and sites of branching morphogenesis in the mouse embryo and placenta. Dev. Dyn.

